# Relationship between the Size of Bent-Shaped Molecules and Mesophase Formation: A Computational Study

**DOI:** 10.1021/acs.jpcb.4c08662

**Published:** 2025-07-07

**Authors:** Piotr Kubala, Wojciech Tomczyk, Michał Cieśla

**Affiliations:** † Institute of Theoretical Physics, Jagiellonian University, Łojasiewicza 11, 30-348 Kraków, Poland; ‡ Institute of Materials Engineering, Faculty of Science and Technology, 49568 University of Silesia, 75 Pułku Piechoty 1A, 41-500 Chorzów, Poland

## Abstract

Mesogenic properties arise from the right conditions imposed by the molecular structure of molecules. Knowledge of how, for example, alterations in molecules length-to-width ratio affect mesophase emergence is of crucial importance. Motivated by ongoing research on the nature of the twist-bend nematic phase and its structural characteristics, we conducted Monte Carlo simulations of bent-shaped molecules represented as a rigid set of *n* hard tangent spheres evenly attached along the arc. With a fixed bend angle, we have identified a set of mesophases in the domain of variable *n* and packing fraction η, among which are twist-bend nematic, splay-bend smectic, and type 1 columnar banana phase—for the first time being reported in a computational study.

## Graphical abstract

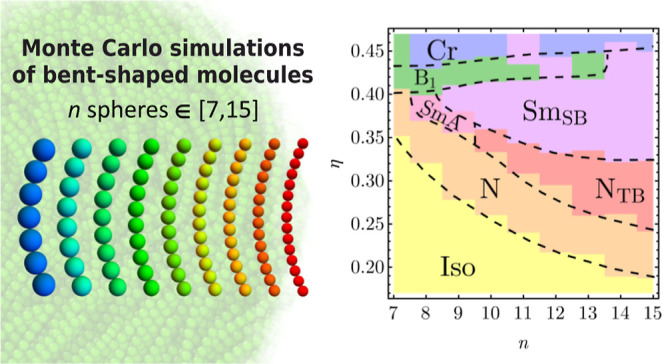

## Introduction

Liquid crystals (LCs) constitute a notable intermediate state of matter between the liquid and the crystal, possessing the properties of both.^1^ The simplest among various liquid crystalline phases is the nematic (N). This mesophase is the result of the self-assembly of anisotropic molecules, which tend, on average, to align along the preferred direction, termed as a “director” (n̂) . Additionally, the molecules’ centers of mass are distributed randomly over the sample volume. Preserving the latter feature and simultaneously inducing periodicity in **n̂** by adding position dependence leads to a broad family of nematic modulated structures of various complexity.^2–4^ For example, in chiral nematic, the director field forms a helix, whereas in blue phases, the director field with double twist is made up of tubes stacked against each other to generate cubic lattices or amorphous systems stabilized by disclinations (linear defects). Over the years, a variety of novel modulated structures have been predicted theoretically (see, e.g., refs 5–14 ), several of which have been confirmed experimentally;^15–20^ one of them is twist-bend nematic (N_TB_).

A remarkable hallmark of N_TB_ (Figure 1) is that this chiral and locally polar phase is formed primarily in systems composed of achiral mesogens. It constitutes the first example of spontaneous mirror symmetry breaking (SMSB) in a liquid state without support from long-range spatial ordering.^21^ This has far-reaching implications that manifest in the form of a heliconical (modulated in one dimension only) structure of a nanoscale pitch with a ground state characterized by a degenerate sign of chirality (so-called “ambidextrous chirality”), resulting in a coexistence of helices with both handednesses: left and right. Peculiar also is the behavior and hierarchy of elastic constants in a nematic phase (see, e.g. refs 22 and 23 ) that precedes[Fn fn1] N_TB_ upon cooling from an isotropic phase. In no time, N_TB_ became a prominent subject of attention within the community of liquid crystal researchers around the world^25^—however, the terminology of this novel phase is not unanimous. The small pitch and sizable polar order were used by Samulski et al.^26^ as an argument to challenge the commonly accepted view that the experimentally observed phase is N_TB_. Instead, they suggested to call it a polar twisted nematic (N_PT_). Dozov and Luckhurst later elucidated this ambiguity^27^ by showing that N_PT_ has the exact symmetry as N_TB_; thus it falls within the set of N_TB_ theories. Hereafter, we will continue to use the “twist-bend nematic” term to conform to our previous work.^39^

**Figure 1:**
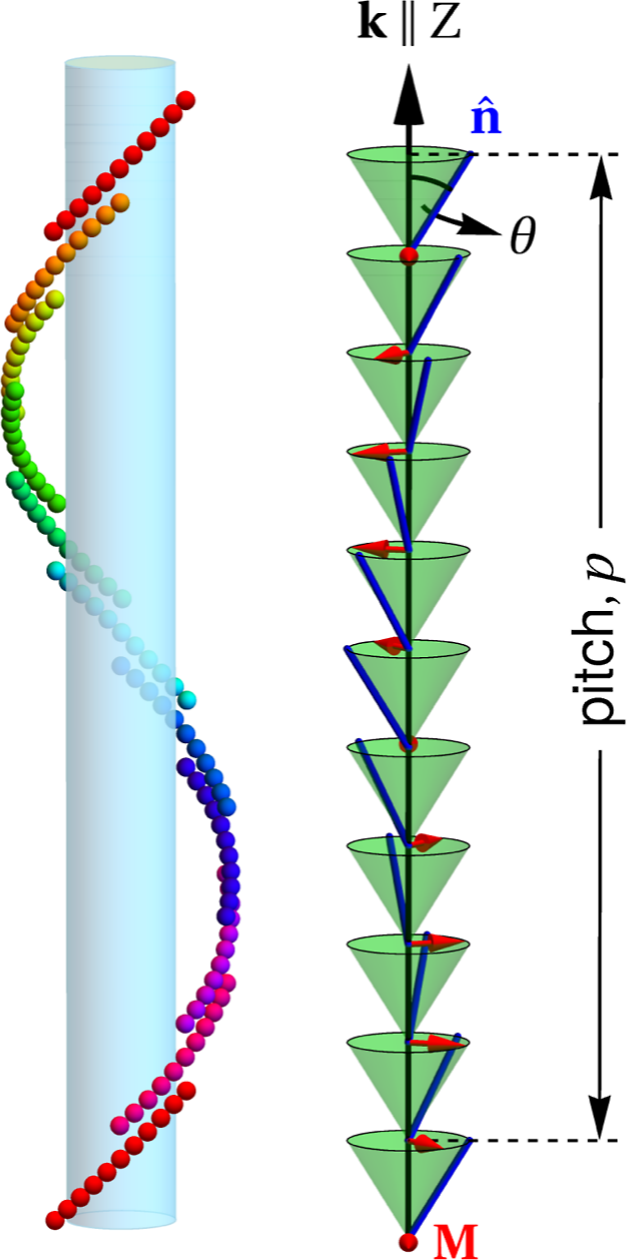
Heliconical arrangement (one of the two degenerate domains) of achiral, bent-shaped molecules in the twist-bend nematic phase. Key aspects of N_TB_ comprise: finite pitch *p* (usually a few nanometers); tilt angle θ (0 < θ < π/2) between the director **n̂** and helical axis k∥Z⁡(k=(2π/p)ẑ) ; the polarization M⁡(M∥n̂×k) is precessing around **k** along with **n̂**; lack of density modulation.

Today an immense number of compounds are known to exhibit the N_TB_ phase, i.a. dimers,^15–17,28–32^ trimers, tetramers, and higher oligomers,^33,34^ along with hybrid bent-core LC^35^ and hydrogen-bonded oligomers.^36^ In addition, some N_TB_-forming mixtures were reported.^29,37^ All that is owed to Meyer’s^6^ and Dozov’s^7^ conjecture supported by computer simulations,^8,14,39,38^ that the properly “curved” (bent) shape of the molecules is sufficient to stabilize[Fn fn2] the N_TB_. However, some aspects of the structure of the molecules still merit a thorough examination, such as how the size of the molecules affects the stability of N_TB_. In that context, very famous are CB*n*CB dimers [the 1,ω-bis­(4-cyanobiphenyl-4′-yl) alkane homologous series; for chemical structure see Figure 2b], where solely the odd members form N_TB_.

**Figure 2:**
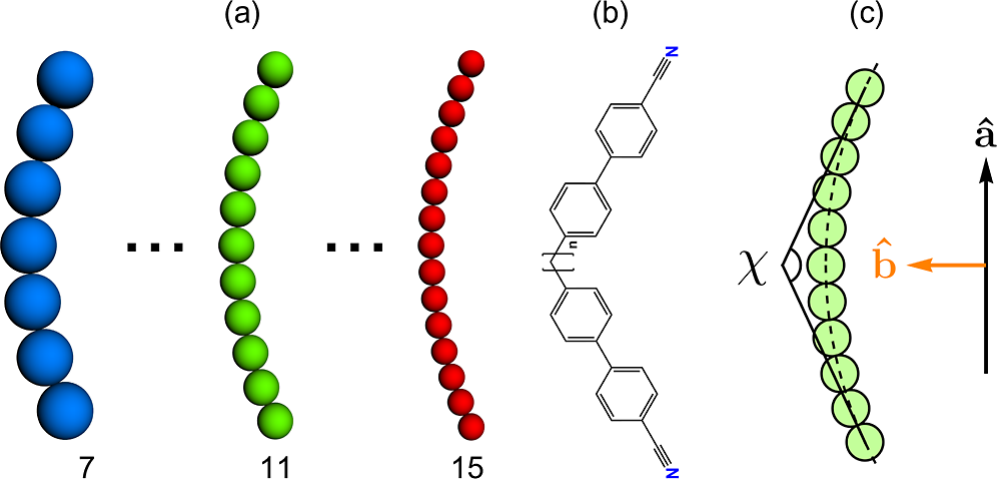
(a) Tangent *n* spheres, where *n* ∈ [7, 15], with fixed curvature being defined in (c). (b) Chemical structure of CB*n*CB homologues series where “CB” stands for “cyanobiphenyl” and “*n*” the number of methylene units in the spacer. (c) Schematic representation of our tangent hard-sphere molecule model with *n* = 11; χ denotes the bend angle, **â** is the long molecular axis, and **b̂** is the short molecular axis (parallel to polarization axis).

Generally, several phenomena are related to the homologous mesomorphic series, e.g. the existence of an odd–even effect,^51^ the (de)­stabilization of the mesomorphic properties^52,53^ and the tendency to form nematic or smectic phases.^52–54^ Moreover, for dimers, one can also observe the appearance of interdigitated and intercalated smectic phases or counterintuitive behaviors such as the promotion of the nematic phase with an increase in the length of molecules.^51^

## Model Systems and Computational Methods

### Molecular Model

Each molecule is composed of *n* tangent spheres of diameter σ placed on a circular arc^39,38^ (Figure 2a) to mimic the curved shape of the N_TB_-forming mesogens (Figure 2b). The measure of the arc’s curvature, designated onward as the molecule’s bend angle χ, is defined as the angle formed by two lines, tangent to the arc at the centers of end-point spheres (Figure 2c). Here, we investigate the stability–structure dependence, initiated in ref 39, by varying the length of the molecules (*n* ∈ [7, 15]) either by adding or subtracting the following spheres while maintaining a constant value of χ. This approach can be associated with attaching/detaching subsequent functional groups to the dimers spacer (see, e.g., refs 44, 55, and 56 and the references therein). All simulations were conducted with a fixed χ = 130°, and the choice of this particular value can be justified as follows.

The research on the twist-bend nematic phase has shown that its stability is significantly dependent on χ. Although the values of the bend angle should be considered as averaged qualitative characteristics, it has been widely acclaimed that N_TB_ becomes stable when χ is roughly between 110°–150° (see, e.g., refs 3, 38, 41, 43, 49, 57–62 ).

These references collectively underscore the subtle contribution of adequate mesogen curvature, i.e., bend angle, in N_TB_ formation with some exceptions that only prove the rule (see, e.g., refs 63, and 64 ). Furthermore, these studies contextualize the choice of 130° in our research as a practical midpoint, which was also dictated by the fact that it constitutes a suitable benchmark for reference with previous computational works.^14,39,38,43,57,59,60,65^ Additionally, this well-documented midpoint can be extended for future parameter-dependent exploration. For example, if one were to study other bend angles (which merits future investigations), it would undoubtedly impact the phase diagram, as it would now be 3-dimensional, i.e, η­(*n*, χ). This constitutes a major endeavor that could lead to structures similar to those reported by Subert et al.^66^

The entropy-driven formation of liquid crystalline phases has been extensively studied for rigid bodies.^67^ Thus, we focus on a hard-sphere model, where the interaction energy is zero if the constituent spheres are disjoint, while for overlapping spheres the energy is infinite.^68^ Consequently, the only independent parameters for a fixed angle χ are the number of spheres *n* and the packing fraction η.

We define the packing fraction η as the ratio of the total volume of spheres building the molecules and the volume of the simulation box

1 $$\eta =\frac{NV_{m}}{V_{b}}=\frac{N\cdot n\cdot \pi \sigma /6}{\left|\left(v_{1}\times v_{2}\right)\cdot v_{3}\right|}$$

where *N* is the number of molecules in the system, σ is the diameter of the sphere, and **v**_*i*_, *i* = 1, 2, 3 are the vectors that span the simulation box. It should be noted that the effective volume of a molecule, *V*_m_^eff^, is slightly higher due to inaccessible regions near the tangent points of the spheres. Assuming *V*_m_^eff^ is given by the maximal superset containing the molecule that preserves the overlap criterion, the gain is equal to

2 $$\frac{V_{m}^{eff}-V_{m}}{V_{m}}=\frac{n-1}{n}\left(\frac{2}{3}-\frac{\pi \sqrt{3}}{4}\right)\approx 0.14\cdot \frac{n-1}{n}$$

### Computer Simulations

We performed constant pressure (*NPT*) Monte Carlo (MC) simulations^69^ for all *n* between 7 and 15 (Figure 2a), with N = 7200 for *n* ∈ [7, 9] and N = 4800 for *n* ∈ [10, 15] in a box with periodic boundary conditions (PBC). For hard-core interactions, the only independent parameter is the ratio of pressure and temperature *P*/*T*. Furthermore, one defines the reduced ratio P*/T* = *pV*_m_/*k*_B_ *T*, which is dimensionless and gives the same packing fraction independent of the molecule’s volume *V*_m_. To scan various packing fractions between η ≈ 0.2 and η ≈ 0.5, each system was simulated under various ratios in the range P*/T* ∈ [1, 10]. The initial configurations were slightly diluted antiferroelectric hexagonal crystals with η ≈ 0.40, with six layers for *n* ∈ [7, 9] and four layers for *n* ∈ [10, 15]. More precisely, in a single layer, the mass centers were arranged in a hexagonal honeycomb pattern, and the orientations of the molecules were identical, with **â** perpendicular and **b̂** parallel to the layer; adjacent layers were identical, except for the opposite sign of the polarization axes b̂↔−b̂ . The initial configuration was then thermalized for 10^8^ MC cycles under P*/T* = 8 – 10 to retain the original packing density η ≈ 0.40. Such equilibrium systems were then used as the starting point for the rest of the simulations, with the system being compressed or expanded to all target densities in parallel. The equilibration was slow due to the concavities of the model molecule, which required 5 × 10^7^ to 5 × 10^8^ MC cycles. The production (averaging) phase lasted at least 5 × 10^7^ cycles.

Each MC cycle consisted of N molecular rototranslation moves and a single box move. The molecular move consisted of arbitrarily selecting a molecule, translating it by a random vector, and rotating it around a random axis by a random angle. If the move did not introduce an overlap, it was accepted; otherwise, it was rejected. The box move was made by performing an appropriate box deformation and accepting it according to the Metropolis-Wood criterion.^69–71^ The smectic phases were simulated in a general triclinic box in which all components of the box vectors were perturbed. In contrast, nematic and isotropic phases were simulated in an orthorhombic box, where only the side lengths were altered. To minimize the probability that the obtained states are metastable or forced by the dimensions of the box, especially those with high density, we performed additional simulations of larger systems (up to N = 19,200). Several different initial conditions were also taken into account. The additional simulations confirmed the consistency of the results.

### Order Parameters and Correlation Functions

For the purpose of quantification and description of the observed phases, we have employed a set of order parameters and correlation functions. Nematic order can be computed using the second-rank order parameter^1^

3 $$P_{2}=\frac{1}{N}\sum_{i=1}^{N}P_{2}\left({â}_{i}\cdot \mathrm{n̂}\right)$$

where the sum is over all N molecules in the system, *P*_2_(···) is the Legendre polynomial of degree 2 and ${â}_{i}$ is a molecular axis vector of the *i*th molecule (cf. Figure 2c).

In general, if the director **n̂** is not assumed a priori, the instantaneous value of *P*_2_ for a single snapshot can be computed using the **Q**-tensor^1^

4 $$Q=\frac{1}{N}\sum_{i=1}^{N}\frac{3}{2}\left({â}_{i}⊗{â}_{i}-\frac{1}{3}1\right)$$

where the sum goes over all molecules in the system. *P*_2_ is determined as **Q**-tensor’s nondegenerate eigenvalue with the largest modulus, and its corresponding eigenvector is the director **n̂**.^13^ Finally, it is averaged over many system snapshots for better statistics (ensemble averaged value will be denoted as ⟨*P*_2_⟩). For an ideal nematic, where all long molecular axes **â** are mutually parallel, ⟨*P*_2_⟩ = 1, while for an isotropic liquid: ⟨*P*_2_⟩ = 0.

Layer formation may be probed using the smectic order parameter^1^

$$\left\langle \tau \right\rangle =\left\langle \frac{1}{N}\left|\overset{N}{\underset{i=1}{\sum }}\exp\left(ik\cdot r_{i}\right)\right|\right\rangle$$ 5

where ⟨···⟩ denotes ensemble averaging, i is the imaginary unit, **k** is the wavevector and **r**_*i*_ is the position of the mass center of the *i*th molecule. Please note that the modulus |···| is taken before ensemble averaging to accommodate a diffusive drift of the smectic order parameter’s complex phase. For a disordered system ⟨τ⟩ = 0, while for perfectly layered one ⟨τ⟩ = 1. The wavevector **k** must be compatible with periodic boundary conditions; thus, its possible values may be enumerated using Miller indices^72^

6 $$k_{hkl}=hg_{1}+kg_{2}+lg_{3}$$

where **g**_*i*_ are reciprocal box vectors. One should select *hkl* indices giving the highest ⟨τ⟩ value. As the systems are initially prepared in a layered configuration, we fix *hkl* at 004 or 006, depending on the number of layers.

The tendency to form a hexagonal honeycomb structure within the layers may be detected by using the local hexatic bond order parameter. In 2D, it is defined as

$$\left\langle \psi_{6}\right\rangle =\left\langle \frac{1}{N}\overset{N}{\underset{i=1}{\sum }}\frac{1}{6}\left|\overset{6}{\underset{j=1}{\sum }}\exp\left(6i\theta_{ij}\right)\right|\right\rangle$$ 7

The inner summation goes over six nearest neighbors of the *i*th molecule, and θ_*ij*_ is the angle between the vector joining the *i*th and *j*th molecule and an arbitrary direction.[Fn fn3] It should be noted that the hexatic bond order parameter is usually defined globally, with the modulus enclosing both sums. Then, it can detect long-range bond order in phases such as type B or type I smectic,^1^ where local bonds retain the same direction throughout the whole system. However, we focus only on local ordering, which is reflected in eq 7. To generalize the definition to three dimensions, we project molecules’ mass centers onto the nearest layer plane and proceed with the 2D formula. The maximum value of ⟨ψ_6_⟩ is 1 for a perfect hexagonal honeycomb structure, while for a disordered smectic ⟨ψ_6_⟩ ≈ 0.37. To extend the procedure to nonlayered phases, the simulation box is artificially partitioned into four or six layers (in consonance with **k**_*hkl*_).

Quantifying the local and global ordering of the polarization axes of molecules **b̂** is also essential. The results show that the polarization vector, otherwise constant in a plane, can change when moving in the orthogonal direction (wavevector **k**). Thus, we measure the planar polarization as follows

$$\left\langle M\right\rangle =\left\langle \frac{1}{N}\overset{n_{L}}{\underset{l=1}{\sum }}∥\underset{i\in layerl}{\sum }{\mathrm{b̂}}_{i}∥\right\rangle$$ 8

The system is divided into layers of *n*_L_. It should be as high as possible (for N, *n*_L_ → ∞ and N^1/3^ *n*_L_^–1^ → 0, the sum changes into the integral along the wavevector), as long as the number of molecules within the layer is large enough to preserve good statistics. We chose *n*_L_ = 50 as a compromise. The outer sum $\sum_{l=1}^{n_{L}}\cdot \cdot \cdot$ goes over all *n*_L_ layers, while the inner sum ∑_*i*∈layer *l*_··· iterates all molecules within the *l*th layer and ∥···∥ is the standard *L* ^2^ norm of a vector. Planar polarization ⟨M⟩ reaches 1 in a maximally polarized system and vanishes in a nonpolar one.

To compute the local polarization, we define

$$\left\langle m^{\left(r\right)}\right\rangle =\left\langle \frac{1}{N}\overset{N}{\underset{i=1}{\sum }}\frac{1}{r+1}∥\underset{j\in rNN,i}{\sum }{\mathrm{b̂}}_{j}∥\right\rangle$$ 9

where the inner sum goes over the set with the *i*th particle and its *r* nearest neighbors. By inspection of multiple values, we found that *r* = 9, corresponding to coarse-graining radius of around two sphere diameters, gives a good compromise between statistics and locality, and we will write ⟨*m* ^(9)^⟩ ≡ ⟨*m*⟩. It should also be noted that different choices of *r* lead to similar qualitative results. Local polarization ⟨*m*⟩ reaches 1 for a maximally polarized system and, for *r* = 9, falls to ⟨*m*⟩ ≈ 0.36 in a nonpolar system. It is important to note that ⟨*m*⟩ does not correspond to the average modulus of the polarization field: ⟨*m*⟩ ≠ *V* ^–1^∫d^3^ *x* ∥**M**∥. However, growing ⟨*m*⟩ qualitatively signals the onset of a nonzero local polarization in the system, while its value roughly measures the relative polarization’s strength.

Further insight into the observed mesostructures can be provided by a carefully selected set of pair correlation functions. The *S*-expansion functions^73^ were shown to be a robust indicator of director and polarization field modulations.^39^ We will use the following three correlation functions to measure the variation in the direction of the system’s wavevector^73^

10 $$S_{aa}^{220}\left(\Delta z\right)={\left\langle \frac{3}{2}{\left({â}_{i}\cdot {â}_{j}\right)}^{2}-\frac{1}{2}\right\rangle }_{ij}$$

11 $$S_{bb}^{110}\left(\Delta z\right)={\left\langle {\mathrm{b̂}}_{i}\cdot {\mathrm{b̂}}_{j}\right\rangle }_{ij}$$

12 $$S_{aa}^{221}\left(\Delta z\right)={\left\langle \left[\left({â}_{i}\times {â}_{j}\right)\cdot {ẑ}_{ij}\right]\left({â}_{i}\cdot {â}_{j}\right)\right\rangle }_{ij}$$

where ${â}_{i}$ and ${\mathrm{b̂}}_{i}$ are, respectively, long molecular and polarization axes of molecule *i*, ⟨···⟩_*ij*_ is averaging over all pairs of molecules with their *z* coordinates differing by around Δ*z* and ${ẑ}_{ji}$ is the **z**-axis versor pointing from molecule *j* to molecule *i*. The first function *S*_aa_^220^(Δ*z*) measures nematic-like correlation, the second one *S*_bb_^110^(Δ*z*) quantifies polar correlations, while the last one *S*_aa_^221^(Δ*z*) indicates whether there is chirality in the system.

## Results

Within the analyzed molecular model, the following phases were observed:^4^ isotropic (Iso), nematic (N), twist-bend nematic (N_TB_), smectic A (SmA), splay-bend smectic (Sm_SB_) and type 1 banana phase (B_1_). The phase diagram in (*n*, η) parameters is shown in Figure 3, while system snapshots are gathered in Figure 4 (nematic phases), and Figure 5 (smectic phases).[Fn fn4] Finally, the order parameters as a function of (*n*, η) are plotted in Figure 6.

**Figure 3:**
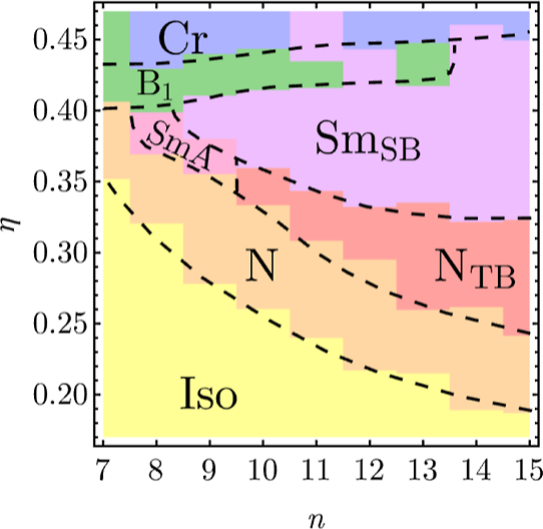
Phase diagram as a function of spheres number *n* and packing fraction η. The following phases are present: isotropic (Iso), nematic (N), twist-bend nematic (N_TB_), type A smectic (SmA), splay-bend smectic (Sm_SB_), type 1 banana phase (B_1_). Around η ≈ 0.44, the system crystallizes; crystalline structure identification is beyond the scope of this manuscript.

**Figure 4:**
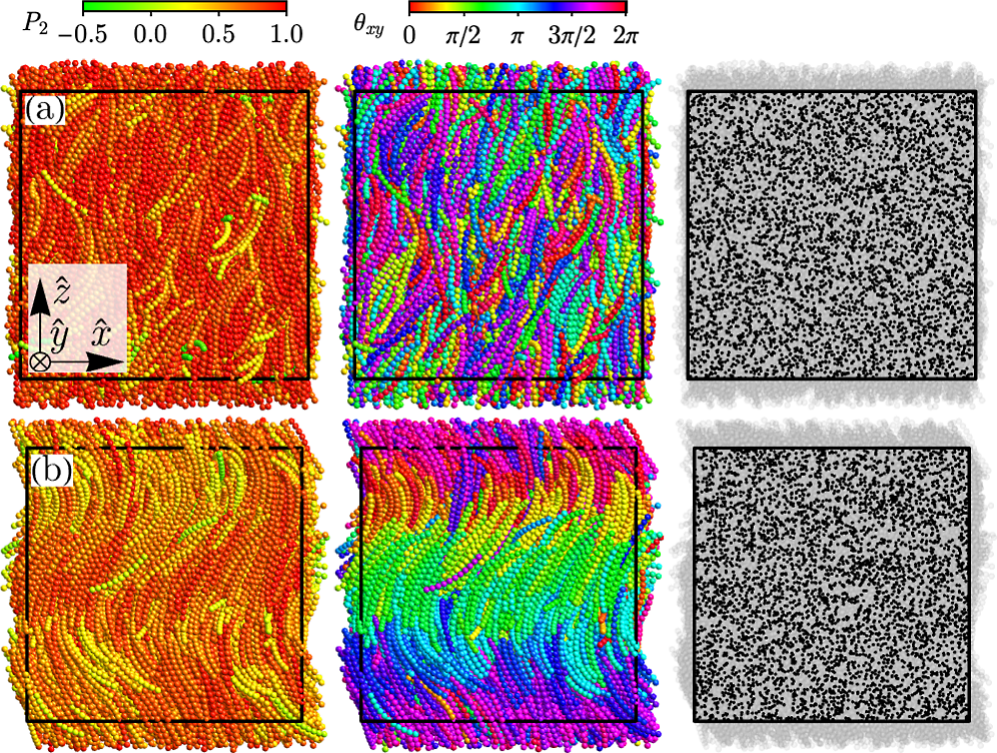
System snapshots of nematic phases: (a) nematic N (*n*, η) = (10, 0.29), and (b) twist-bend nematic N_TB_ (*n*, η) = (11, 0.33). Each panel in a single row corresponds to the same snapshots with different features highlighted: the first column is color-coded according to nematic parameter $P_{2}\left({â}_{i}\right)=3/2\left[{\left({â}_{i}\cdot \mathrm{n̂}\right)}^{2}-1/3\right]$ , the second one according to counterclockwise angle θ_*xy*_ between *xy* projection of polarization axis ${\mathrm{b̂}}_{i}$ and **x̂** versor. At the same time, the last one shows molecules mass centers depicted as black dots.

**Figure 5:**
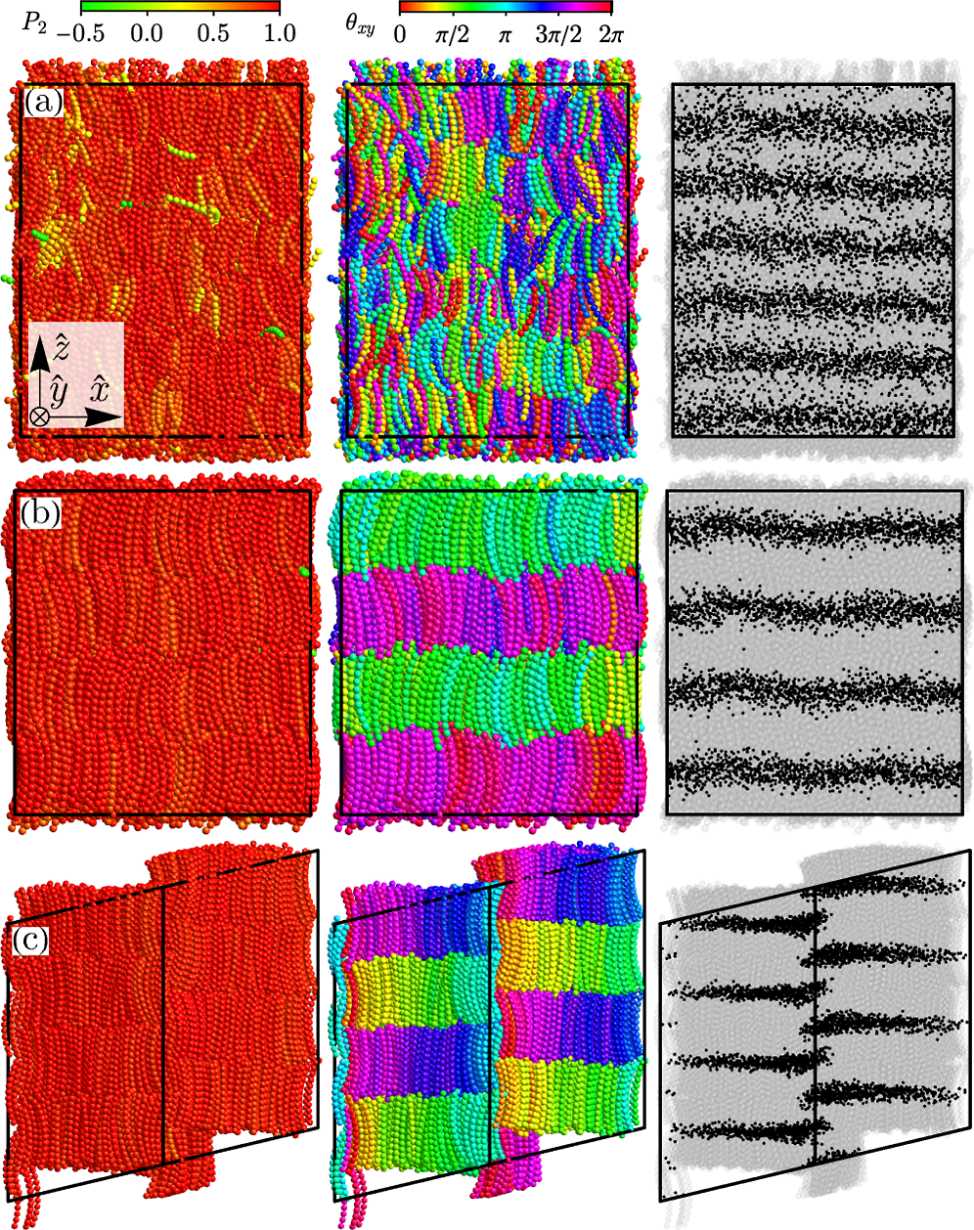
System snapshots of smectic phases: (a) type A smectic SmA (*n*, η) = (8, 0.37), (b) splay-bend smectic Sm_SB_ (*n*, η) = (10, 0.40) and (c) type 1 banana phase B_1_ (*n*, η) = (11, 0.43). Each panel in a single row corresponds to the same snapshot with different features highlighted (see Figure 4). The perspective in panel (c) is such that two edges of the periodic triclinic simulation box coincide into a single segment (the middle vertical one).

**Figure 6:**
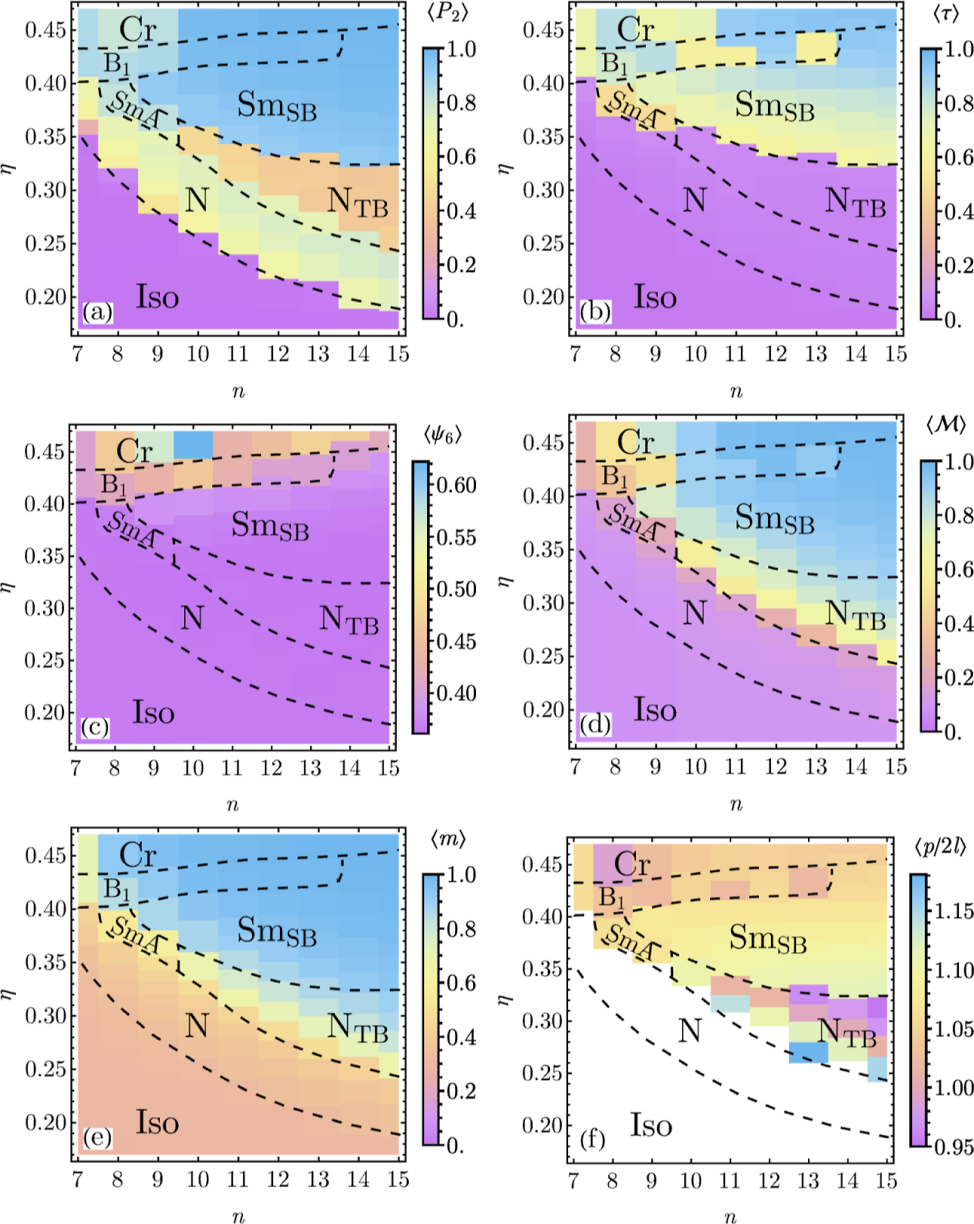
Order parameters and system period as a function of the number of spheres *n* and packing fraction η: (a) nematic order ⟨*P*_2_⟩, (b) smectic order ⟨τ⟩, (c) local hexatic bond order ⟨ψ_6_⟩, (d) planar polarization ⟨M⟩ , (e) local polarization ⟨*m*⟩ and (f) ratio of half system period and end-to-end molecules’ length *p*/2*l*.

The nematic phase is present for all studied values of *n*; however, the Iso-N phase boundary rises quickly with decreasing *n*—from η = 0.19 for *n* = 15 to η = 0.35 for *n* = 7 (Figure 3a). In the whole range, all order parameters, except ⟨*P*_2_⟩, have low values (Figure 6). On the other hand ⟨*P*_2_⟩ is high, in the range between [0.5, 0.8], which is similar to other computational studies of hard-core shapes.^39,74–76^ However, it should be noted that in experiments ⟨*P*_2_⟩ is usually slightly lower, that is, between 0.4 and 0.7.^77^ The ⟨*P*_2_⟩ is approximately constant on the lower and upper nematic phase boundaries and increases with the packing fraction η. We also observe a slight increase in local polarization ⟨*m*⟩ near the upper boundary from the disorder value ⟨*m*⟩ ≈ 0.36 to ⟨*m*⟩ ≈ 0.45, which is expected since the N_TB_ phase is locally polar (as will be discussed in the next paragraph). The nematic phase range in the packing fraction is near constant with Δη ≈ 0.05.

Twist-bend nematic phase is characterized by the director field that simultaneously twists and bends in space by precessing on the side of a circular cone that is coaxial with the **z**-axis of the laboratory reference frame (see Figure 1). As a result, a heliconical structure can form either right- or left-handed as a result of the SMSB. The director field modulation can be well described by

$${\mathrm{n̂}}_{p,\theta }\left(x,y,z\right)=\left(\begin{array}{c} \sin\left(\theta \right)\cos\left(2\pi z/p\right)\\ \sin\left(\theta \right)\sin\left(2\pi z/p\right)\\ \cos\left(\theta \right) \end{array}\right)$$ 13

Here, *p* = 2π/*k* is the pitch, while θ is the tilt angle (half of the opening angle of the cone). Polarization is orthogonal to both **n̂**(*x*,*y*,*z*) and *k*∥ ***z*** *^*

$$M_{p}\left(x,y,z\right)=M\left(\begin{array}{c} \sin\left(2\pi z/p\right)\\ -\cos\left(2\pi z/p\right)\\ 0 \end{array}\right)$$ 14

In this case, the polarization modulus *M* is proportional to the planar order parameter ⟨M⟩ as defined in eq 8.

We observe that for *n* ≥ 10 and with increasing density, N_TB_ is preceded by the nematic phase (Figure 3). Lower and upper phase boundaries of N_TB_ fall with the growing *n*; on the other hand, the range in packing fraction increases—it is η ∈ [0.34, 0.37] for *n* = 10, and η ∈ [0.24, 0.32] for *n* = 15. Near-zero ⟨τ⟩ values (Figure 6b) and high ⟨*P*_2_⟩ values (Figure 6a) confirm that it is nematic. The nematic order parameter is almost constant in the entire range with ⟨*P*_2_⟩ ≈ 0.40. Importantly, we observe the onset of the local ⟨*m*⟩ (Figure 6e) and planar ⟨M⟩ (Figure 6d) polarization order; the first spans from ⟨*m*⟩ ≈ 0.50 at the lower boundary to ⟨*m*⟩ ≈ 0.80, while the second spans from ⟨M⟩≈0.30 to ⟨M⟩≈0.75 .

Solid evidence for N_TB_ is given by the inspection of the numerically obtained director profile[Fn fn5] in Figure 7a, where its {*x*, *y*, *z*} components are plotted against the *z*–coordinate within the simulation box. The *n*_*z*_ coordinate remains constant, while *n*_*x*_ and *n*_*y*_ are sinusoidal with the same amplitude and dephased by a quarter of the period, which confirms that the director is precessing around the cone along the structure’s wavevector. Figure 6f shows how *p* varies with η and *n*. Likewise for order parameters, the period is nearly constant on both the phase boundaries of Iso-N_TB_ and N_TB_-Sm_SB_ phase boundaries and is close to two molecular lengths[Fn fn6] 2*l*. However, it decreases slightly with compression, with *p*/2*l* ≈ 1.15 on the lower boundary to *p*/2*l* ≈ 0.95 on the upper boundary. The tilt angle θ can be obtained by fitting the relation (13) to the numerical data. The variability of θ is very low—for all simulation points, it lies in the range θ ∈ [33.5°, 39°]. Amazingly, this result coincides well with the experimental outcomes, theoretical predictions, and all-atom simulations (see, e.g. refs 23, 57, 59, 60, 65, 79, and 80 ).

**Figure 7:**
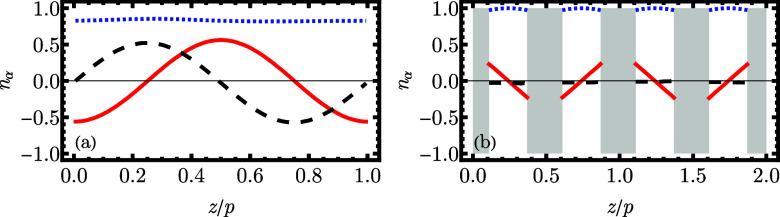
Components *n*_α_ (α = *x*, *y*, *z*) of the director **n̂** as a function of the *z* coordinate (along the wavevector **k**) for (a) N_TB_ (*n*, η) = (11, 0.33), and (b) Sm_SB_ (*n*, η) = (11, 0.40), computed from the numerical simulations. The subsequent components are plotted as α = *x* red solid line, α = *y* black dashed line, and α = *z* blue dotted line. The gray areas in panel (b) correspond to regions between the layers with hardly any molecules.

Additional information is retrieved from the analysis of the correlation functions (Figure 8). In all three panels, black and red dashed lines refer to the N_TB_ phase for, respectively, a shorter molecule with *n* = 11 and a longer one with *n* = 15. Both cases are qualitatively similar. However, the amplitudes are more significant for *n* = 15, which hints that orientational fluctuations around the director are more damped with growing *n*. The *S*_aa_^220^ correlation function (Figure 8a) measuring nematic-like correlations falls well below zero for Δ*z* = *p*/2 when the directors lie on the opposite side of the precession cone. The *S*_bb_^110^ correlation function (Figure 8b), which probes the polarization, follows the oscillating pattern [cf. eq 14]. Finally, it can be stated that the system is chiral based on the *S*_aa_^221^ correlation function (Figure 8c).

**Figure 8:**
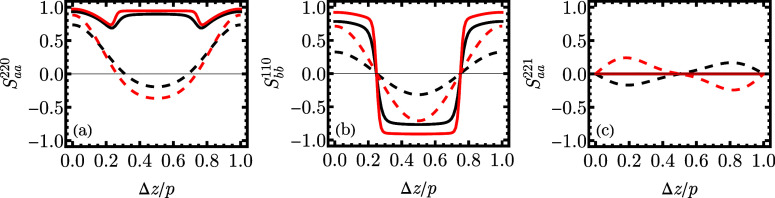
Correlation functions *S*_αα_^ijk^ for shorter molecule with *n* = 11: N_TB_ η = 0.32 (black dashed line), Sm_SB_ η = 0.37 (black solid line) as well as for longer molecule with *n* = 15: N_TB_ η = 0.29 (red dashed line), Sm_SB_ η = 0.39 (red solid line). The subsequent panels show (a) *S*_aa_^220^, (b) *S*_bb_^110^, and (c) *S*_aa_^221^ correlation functions against the ratio of distance along the *z* axis and structure’s period.

Despite the applied simplifications and limitations arising from the incorporated hard-sphere model, we could qualitatively grasp all characteristic features of N_TB_. Our results rank well with respect to the values of ⟨*P*_2_⟩ and the tilt angle of N_TB_, although they are relatively closer to the upper limits of the experimental reports, which is not surprising. The general tendency, based on the literature, is that the *S* ≡ ⟨*P*_2_⟩ ∈ [0.35, 0.45] (e.g. refs 81, and 82 ) and the heliconical tilt angle 20° ≲ θ ≲ 35° (e.g. refs 79, 80, 83, and 84 ), but it should be emphasized that these values depend on the research method used. Incorporating flexibility, for example, through soft potentials or dynamic linkers, could even better mimic experimental conditions.

Onward, for *n* = 8 and 9, the N_TB_ phase is replaced by SmA as indicated by high ⟨*P*_2_⟩ and ⟨τ⟩, along with low ⟨M⟩ and moderate ⟨*m*⟩ values, as seen in Figure 6, as well as by visual inspection of the system’s snapshot (Figure 5a). Director modulation is not observed. Local polarization correlations are visible, as confirmed by ⟨*m*⟩ ≈ 0.5, which is a value higher than that of the nonpolar Iso and N phases but lower than the rest of the polar phases. On the other hand, near zero ⟨M⟩ excludes the presence of a long-range polar order.

Directly above N_TB_ and SmA, a polar smectic is observed, as indicated by high values of all the order parameters of ⟨*P*_2_⟩, ⟨τ⟩, ⟨M⟩ , and ⟨*m*⟩. The phase is present for *n* ∈ [9, 15] and its range widens from Δη ≈ 0.03 for *n* = 9 to Δη ≈ 0.13 for *n* = 15. System snapshot (Figure 5b) reveals that the phase is antipolar (antiferroelectric). The director averaged over the entire layer is constant and orthogonal to the layers, and the direction of the averaged polarization is also constant but parallel to the layers; however, at the same time, its sense alternates. In the literature, this phase is denoted as the antiferroelectric type A smectic (SmAP_A_).^3^ However, the structure in our system is slightly more complicated, with a visible modulation of the director field, as shown in Figure 7b. Notably, there are hardly any molecules between the layers (gray areas in the plot). Thus, the director field is well-defined only in the finite range around the middle of the layer. It is clear that the director performs an oscillatory movement in the *xz* plane and has a nearly zero *y* component. Thus, we will refer to this phase as splay-bend smectic (Sm_SB_).

The director field for splay-bend nematics and smectics can be well described by the following variational *ansatz* ^14^

$${\mathrm{n̂}}_{p,\theta }\left(x,y,z\right)=\left(\begin{array}{c} \sin\left(\theta \right)\cos\left(2\pi z/p\right)\\ 0\\ \sqrt{1-\sin^{2}\left(\theta \right)\cos^{2}\left(2\pi z/p\right)} \end{array}\right)$$ 15

This relation fits well with the simulation data and enables us to establish the oscillation angle θ (the opening angle of the flattened cone). Similarly to the tilt angle in the N_TB_ phase, the variability of θ is relatively low, θ ∈ [14°, 19°]. The width of the layer, corresponding to the half-structure period, is presented in Figure 6f. It is close to the molecule’s length and slowly decreases under compression with *p*/2*l* ≈ 1.12 at the lower boundary to *p*/2*l* ≈ 1.04 at the upper one.

Both eqs 13 and 15 can be generalized to the precession around a deformed cone with all three (twist, splay, and bend) deformations present^14^

16 $${\mathrm{n̂}}_{p,\theta_{x},\theta_{y}}\left(x,y,z\right)=\left(\begin{array}{c} \sin\left(\theta_{x}\right)\cos\left(2\pi z/p\right)\\ \sin\left(\theta_{y}\right)\sin\left(2\pi z/p\right)\\ \sqrt{1-\sin^{2}\left(\theta_{x}\right)\cos^{2}\left(2\pi z/p\right)-\sin^{2}\left(\theta_{y}\right)\sin^{2}\left(2\pi z/p\right)} \end{array}\right)$$

The structure is parametrized by two different opening angles θ_*x*_ and θ_*y*_ for the *x* and *y* axes, respectively. If θ_*x*_ = θ_*y*_ = θ it reduces to the twist-bend modulation, eq 13, while for θ_*x*_ = θ, θ_*y*_ = 0 splay-bend modulation, eq 15, is obtained. Interestingly, Chiappini and Dijkstra^14^ were able to observe an intermediate twist-splay-bend smectic Sm_TSB_ with nonzero θ_*x*_ ≠ θ_*y*_ between N_TB_ and Sm_SB_. Similar to the previous study,^39^ this phase is absent in our system; refining the sampling grid around the N_TB_-Sm_SB_ transition shows that both the order parameters and the packing fraction experience a jump, suggesting that the phase transition may be of the first order.

The correlation functions for shorter molecules (*n* = 11, black solid line) and longer molecules (*n* = 15, red solid line) are shown in (Figure 8). The nematic-like correlation function *S*_aa_^220^ remains high throughout the range, since the local tilt of the director to the wavevector remains low (cf. the oscillation angle θ not exceeding 20°). The maximum in Δ*z* = 0.5*p* is lower than in Δ*z* = 0, *p* since the tilt is inverted in the adjacent layers (separated by 0.5*p*). Minima are observed around Δ*z* ≈ 0.25*p*, 0.75*p*. As high-density areas are rather narrow, these distances correspond mainly due to the correlations between the layers and the space between them containing outliers with anomalous orientations. Consequently, this reduces the values of *S*_aa_^220^. The polarization correlation function *S*_bb_^110^ shows strong correlations between an even number of layers and strong anticorrelations between an odd number of layers. Unlike N_TB_, it resembles more a square wave than a sinusoidal one. This is because, for Sm_SB_, the polarization does not revolve around the circle but, like the director, performs an oscillatory movement with a similar and small oscillation angle θ. Finally, *S*_aa_^221^ is zero, indicating that the structure is nonchiral.

As all isotropic, nematic, and smectic states with η < 0.40 were obtained in the simulations by an expansion from a denser system, it is instrumental to check whether compression from a low-density disordered state recreates the same phase sequence. This consistency check was performed for *n* = 11 by gradual compression of an Iso phase near the Iso-N boundary, producing the same Iso–N–N_TB_–Sm_SB_ phases. In Sm_SB_, domains were formed, which prevented the system from arranging into a homogeneous structure as in Figure 5b, presenting the result of an expansion run. Nevertheless, the most important features—layer formation and the tendency for an antiferroelectric arrangement—were retained. No significant hysteresis was observed; it was completely absent for the phase transitions of Iso–N and N–N_TB_, while for N_TB_–Sm_SB_, it was on the order of Δη = 0.01.

One of the unique characteristics of bent-shaped molecules is the enhanced, shape-driven flexopolarization effect, which is a consequence of the coupling between polarization and strain gradient (splay or bend deformation of the director field).^3^ Due to the packing efficiency, especially in the smectic phase, where the molecules are coplanar and their axes parallel, the polarization axes tend to splay.^85^ Consequently, maintaining constant polarization in the *xy* plane in the Sm_SB_ phase involves an increase in the free energy. However, as mentioned before, splay deformation cannot fill the space uniformly without defects. For sufficiently large densities, where the packing efficiency grows in importance, the second mechanism becomes energetically favorable and frustrated structures are formed. In the spirit of this reasoning, we state that the phase presented in Figure 5c is one of such frustrated structures. It has a high nematic and smectic order (Figure 6a,b), as well as a local and global polarization order (Figure 6c,d). Interestingly, the local hexatic order ⟨ψ_6_⟩ is also elevated (Figure 6f). The structure can be identified as part of the type 1 banana phase (B_1_) family.^86–89^ It is characterized by the mesostructure of hexatically packed columns along the **y**-axis (in the coordinate system of Figure 5) with discontinuities in the polarization field between them. The polarization axes are parallel to the **y** axis in the middle of each column. They rotate when one moves away from the center, becoming parallel to the **x**-axis on the boundary. The adjacent columns along the **z** axis prefer antipolar correlations between the molecules—in the middle, the polarizations are antiparallel. It can be attributed to a more compatible vibration motion of the ends of the molecules from the adjacent layers (see Figure 4 in ref 90), which was also the case for Sm_SB_.

The phase occurs in a narrow range of packing fractions Δη = 0.03 above η = 0.40 for *n* ∈ [7, 13], shifting slightly toward higher packing fractions with increasing *n*. The exception is *n* = 12, for which Sm_SB_ instead forms (Figure 3). It is probably a metastable state in contradiction to B_1_, as these two structures are in direct competition. As the B_1_ phase assembles spontaneously when a lower-density Sm_SB_ phase is compressed in a triclinic box, it is doubtful that B_1_ is metastable with respect to Sm_SB_ for all *n*. On the other hand, we cannot pinpoint the actual range of this phase in *n* or η. In particular, the structure borders with three liquid phases: Iso, SmA, and Sm_SB_. To the best of our knowledge, such a structure has never been reported in computational studies. However, similar ones are well-known in experiments.^85,91^ Furthermore, we want to note that the structure was observed in our previous study.^39^ However, we could not assess its stability then; thus, we called it the SmX.

## Conclusions

Using Monte Carlo simulations of constant pressure *NPT* of hard particles, we have analyzed the self-assembly of rigid, bent-shaped molecules with the fixed bend angle of χ = 130° and a varied length that is reflected in the number *n* (*n* ∈ [7, 15]) of adjacent spheres. The stability of the liquid crystalline phases increased with growing *n* pushing all Iso–N-N_TB_-Sm_SB_ boundaries downward. The twist-bend nematic phase, previously reported for the same molecule with *n* = 11,^38,39^ was superseded by the smectic A phase for *n* < 9, pinpointing *n* = 10 as the shortest (thickest) molecule, for which the phase is present in the model. The tilt angles for the N_TB_ and Sm_SB_ structures remained nearly constant in all the corresponding regions and were equal, respectively, around 35° and 17°. For relatively high packing fractions, in the range η ∈ [0.40, 0.43], the frustrated type 1 columnar banana phase was observed. It was constructed from hexatically packed columns with polarization splay within them. To our knowledge, no previous computational works reported a similar phase, although it is well-known from the experiments (see, e.g., refs 85, and 91). This phase is later destabilized for *n* ≥ 14 in favor of Sm_SB_. It should be noted that the molecules that form the twist-bend nematic and banana phases are yet very scarce.^92^

This study, together with our previous one,^39^ helps to understand the importance of molecular curvature and its length (thickness) in stabilizing phases with modulations in director and polarization fields. Future studies may involve further exploration of a similar model with smooth bent spherocylinders instead of multiple spherical beads,^14^ as well as replacing spheres with oblate spheroids or introducing variable chirality at the molecular level.

## Data Availability

The data underlying this study are openly available in the RODBUK Cracow Open Research Data Repository at DOI: 10.57903/UJ/KGZINT. The source code of an original simulation package used to perform Monte Carlo sampling is available at https://github.com/PKua007/rampack.

## Footnotes

1 Mesogens that form N_TB_ directly from the isotropic phase are also known in the literature.^24^

2 Other accompanying factors reported in the literature are conformational distribution,^40–43^ intermolecular interactions,^44–46^ intermolecular torsion,^47–49^ and the effect of the excluded volume.^38,39,50^

3 It is important to note that the director **n̂** computed using such the procedure is a global parameter disregarding any local modulations. To obtain the director profile **n̂**(x,y,z), coarse-grained averaging must be used.

4 Although we identified an approximate boundary of the crystalline phases, structure identification was beyond the scope of this study.

5 The directors were computed from the **Q**-tensors. The latter were averaged over [*z* – δz, *z* + δz] slices and multiple system snapshots, accounting for the movement due to the translational Goldstone mode.^78^

6 For real-life N_TB_-forming mesogens the period, regarding the lengths of the constituent molecules, varies significantly, for example, from 0.25 to 4.^33^
